# Editorial: Assuring trustworthiness of autonomous systems as intelligent and ethical teammates

**DOI:** 10.3389/frobt.2022.1004094

**Published:** 2022-09-26

**Authors:** Siddhartha Bhattacharyya, Meredith Carroll

**Affiliations:** Florida Institute of Technology, Melbourne, FL, United States

**Keywords:** autonomous systems, trust, ethics in autonomous systems, human robot interaction, assurance

Advances in the area of robotics/autonomous systems have significantly increased the prevalence of teaming with robots to accomplish challenging goals. These goals span a diverse set of applications for commercial purposes, across several industries such as agriculture, manufacturing, healthcare, and transportation. Furthermore, with the increased capabilities of artificial intelligence, the potential to allocate responsibility to an autonomous system, or a team of autonomous systems, has increased manifold. Added to this is the emergence of shared autonomy between humans and robotic systems in collaborative tasks. As a result, autonomous systems need to be designed to facilitate the development and maintenance of high levels of human operator trust in, and effective interaction with, autonomous systems to achieve these goals.

Human-robot interaction has been the focus of study for decades. However, it has had a limited impact on the actual design of robotic and autonomous systems. Much of the research related to the design of these systems has focused on which responsibilities to assign to the autonomous system as a team member, without enough emphasis on how human-robot interaction should occur, and the ethical behavior that should be exhibited by the autonomous system. With the increasing complexity of autonomous systems, this interaction plays an increasingly important role in successfully achieving tasks, goals, ethics, handling contingencies, and preventing errors. It becomes especially important when the context dynamically changes, or when dealing with unexpected uncertainty. Furthermore, machine learning technology adds to the complexity of the autonomous system, and as a result, the actions taken by the autonomous system are not obvious or well explained to the human operator. To address this problem, research targeting the effective interaction between human and autonomous system teammates, including the development and maintenance of trust, is being conducted. This special issue contains four articles that aim to elucidate issues associated with trust in human-autonomous system teaming.

In the first article, Lebiere et al. present a computational model of human trust and reliance calibration processes that is grounded in instance-based learning theory and implemented using the ACT-R computational cognitive architecture. The model is developed with empirical data from a simulated human–machine-team in a search and rescue environment requiring participant monitoring and interaction with automation. The authors examine how adding or removing transparency cues, such as the number of sensors collecting data, and disruptions in the relationship between transparency cues and automation behavior, such as reductions in reliability, influence trust and reliance. The findings indicate that both transparency cues and disruptions can influence reliance and that the strength of this relationship depends on prior experience. The authors predict that trust calibration processes could be manipulated to cause overreliance and under-reliance through the use of transparency cues.

In the second article, Van der Waa et al. argue that a human should be responsible for the handling of ethical principles by intelligent agents. To accomplish this, the researchers propose three team design patterns with different levels of autonomy for the agent. They explore the patterns of moral choices made by the agent and by humans for medical triage tasks. The authors evaluate the relevance of the simulation study, the control that a human has over the agent, and the agent’s reasoning by including human understanding. The results indicate that the simulation was realistic. For situations in which the outcomes were quickly noticeable, the human had meaningful control and felt responsible. But, for outcomes that took longer, the control and sense of responsibility seemed reduced. Thus, the authors emphasize the need to include explanations of team roles along with the cognitive state of the human.

In the third article, by Honig and Oron-Gilad, the authors emphasize the need to leverage socio-technical relations between the human and the robot to support the handling of unexpected failures. They explore the use of graceful extensibility to respond to unexpected events. In line with this thinking, the authors propose the design of robots to promote graceful extensibility and allow the Human-Robot Ecosystem to adjust to new contingencies. The authors suggest the expansion from Human-Robot Interaction to Human-Robot Ecosystem, as handling unexpected failure requires more than just a human’s interaction with the robot. It is essential to understand the ecosystem within which the robot executes its operations. As a result, authors focus on a network of socio-technical connections that could solve an unexpected failure as the robot is no longer limited to interaction with one human but a group of humans.

In the final article Rebensky et al. examine the effects that different levels of autonomy (LOA), or the varying levels of responsibility given to the autonomous agents, has on operator trust in the agents in a simulated intelligence surveillance and reconnaissance task. In this study, participants searched for enemy targets with the assistance of four unmanned aircraft vehicle (UAV) teammates to decide the safest route to send a convoy. LOAs were manipulated in four different ways: 1) manual, in which the agent only assisted with target detection; 2) advice, in which the agent detected a target and suggested a classification as enemy or friendly; 3) consent, in which the agent detected and classified the target as friendly or enemy, requiring the participant to confirm the classification; and 4) veto, in which the agent detected, classified and confirmed the targets, with no action required of the participant unless they wanted to veto the agent’s classification. The findings indicate that although performance, stress, and workload levels appear to be more optimal when at the two higher LOAs (i.e., consent, veto), there is no significant difference in trust in the agents between various LOAs. This is potentially due to a range of issues.

The research articles published in this special issue highlight areas of research that are investigating the inclusion of ethics and responsibility allocation to autonomous systems. The articles discuss various methods for optimizing human trust in autonomous systems as their complexity increases.

